# Charon’s refractory factory

**DOI:** 10.1126/sciadv.abq5701

**Published:** 2022-06-17

**Authors:** Ujjwal Raut, Benjamin D. Teolis, Joshua A. Kammer, Caleb J. Gimar, Joshua S. Brody, G. Randall Gladstone, Carly J. A. Howett, Silvia Protopapa, Kurt D. Retherford

**Affiliations:** 1 Center for Laboratory Astrophysics and Space Science Experiments (CLASSE), Space Science and Engineering, Southwest Research Institute, San Antonio, TX 78238, USA.; 2 Space Science and Engineering, Southwest Research Institute, San Antonio, TX 78238, USA.; 3 Department of Physics and Astronomy, University of Texas at San Antonio, San Antonio, TX 78249, USA.; 4 Department of Space Studies, Southwest Research Institute, Boulder, CO 80302, USA.; 5 Department of Physics, University of Oxford, Oxfordshire, UK.

## Abstract

We combine novel laboratory experiments and exospheric modeling to reveal that “dynamic” Ly-α photolysis of Plutonian methane generates a photolytic refractory distribution on Charon that increases with latitude, consistent with poleward darkening observed in the New Horizons images. The flux ratio of the condensing methane to the interplanetary medium Ly-α photons, φ, controls the distribution and composition of Charon’s photoproducts. Mid-latitude regions are likely to host complex refractories emerging from low-φ photolysis, while high-φ photolysis at the polar zones primarily generate ethane. However, ethane being colorless does not contribute to the reddish polar hue. Solar wind radiolysis of Ly-α–cooked polar frost past spring sunrise may synthesize increasingly complex, redder refractories responsible for the unique albedo on this enigmatic moon.

## Teaser

Exosphere model combined with dynamic Lyman-α photolysis experiments reveal the photoproduct distribution at Charon.

## INTRODUCTION

**Fig. 1.**
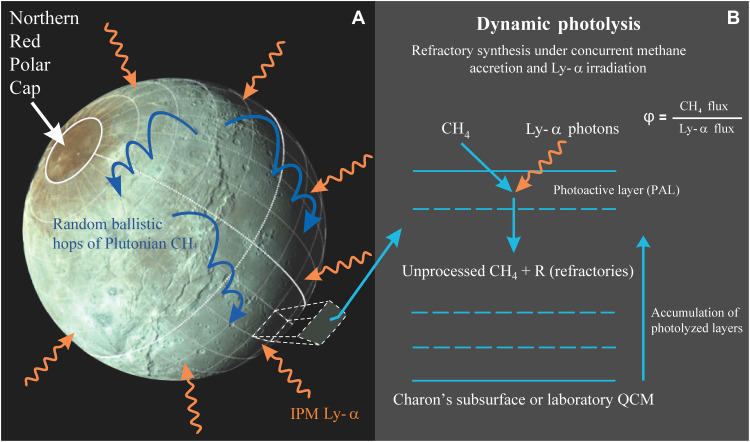
Dynamic photolysis at Charon. (**A**) Charon’s MVIC color image showing the distinct red polar cap region named Mordor Macula nearly circumscribed by the 75° latitude annulus in the sunlit northern hemisphere. The red color is attributed to tholin-like refractories resulting from the photolysis of Plutonian methane by the IPM Ly-α photons (3). Following arrival from Pluto, CH_4_ molecules hop in random gravitationally bound trajectories (blue curves) across Charon’s surface until they are trapped in ultracold locations at polar latitudes in the winter hemisphere. CH_4_ molecules are dissociated by the isotropic IPM Ly-α photons (orange arrows) as they accrete atop Charon’s cold polar surface, exemplified in the extruded zone. (**B**) Simplified illustration of the dynamic photolysis process that converts methane into refractories in the PAL during accretion at cold Charon polar locations, also reproduced in our laboratory experiments. The refractory fraction *RF* in films photolyzed during methane loading strongly depends on φ, the flux ratio of CH_4_ to Ly-α photons. The photolyzed mixture (*R* + unprocessed CH_4_) continues to accumulate on top of Charon’s subsurface until spring equinox when the winter pole emerges into sunlight. The surface temperature rises desorbing the unprocessed methane while leaving the less volatile refractories behind on the polar surface. QCM, quartz crystal microbalance.

The Multispectral Visible Imaging Camera (MVIC) images of Charon taken during the 2015 New Horizons flyby revealed its northern polar region to be marked with a distinct reddish hue (*1*–*4*). This red color appears to be most intense north of 70° latitude (*3*) in the region known informally as Mordor Macula (Fig. 1A), which is nearly circumscribed by an arcuate topographic ridge (*5*). The colocation of the red color within a topographic depression led initially to the speculation of an impact-based origin (*6*). However, Long Range Reconnaissance Imager (LORRI) images of Charon’s southern hemisphere in Pluto-shine also showed poleward darkening, similar to the sunlit northern hemisphere (*3*, *7*). Separate polar impacts needed to explain that the observed darkening at high latitudes on both hemispheres seemed less plausible. Alternatively, Charon’s night side is also continually illuminated by the diffuse Ly-α radiation from the resonant scattering of solar Ly-α by the interplanetary medium’s H atoms. The interplanetary medium Lyman-α (IPM Ly-α) photolyzes the methane cold-trapped onto Charon’s winter pole (following arrival from Pluto) (*8*, *9*) into more complex and less volatile molecules (*3*), precursors to the red-colored tholins following subsequent energetic processing. These processed refractories are also proposed as the cause of the poleward darkening of the visible albedo in the LORRI images (*3*).

To estimate the refractory production rate at Charon, Grundy *et al.* (*3*) made several simplifying assumptions related to methane accretion and photolysis. First, they adopted a uniform and time-independent methane accretion rate (~3 nm per Earth year) above an ad hoc cold pole boundary at 45°, while noting that the CH_4_ accumulation would presumably vary with latitude, being thicker at the poles. Second, they estimated that ~21% of all methane condensed onto Charon undergoes photolysis to produce the red color–imparting refractories, based on the 1.4 × 10^−21^ m^2^ photoabsorption cross section of an unshielded methane molecule (*10*). IPM Ly-α photolysis of a uniform methane frost would generate a homogeneous refractory crust above 45° latitude. In contrast, the MVIC image shows the red material to be concentrated above ~70° with a steady decline in redness toward lower latitudes [Fig. 1A; see also the studies of Protopapa *et al.* (*11*) and Howett *et al.* (*12*)]. Here, we build on the model of Grundy *et al*. (*3*) to reassess the contribution of IPM Ly-α to Charon’s refractories while making two notable advances through implementation of (i) a Charon exosphere model to obtain time- and position-dependent methane accretion rates and (ii) “dynamic” photolysis experiments to quantify the photoconversion rates under Charon-like conditions of concurrent Ly-α irradiation and methane accretion.

The exosphere model constrains the total methane feedstock available for photoconversion at all Charon surface locations. However, what processes control the refractory synthesis in the methane frost that is photolyzed during accrual? Charon’s dynamic photolysis conditions differ fundamentally from the previous experiments (*13*–*18*) that report photolysis cross section and refractory fractions in methane films deposited to “constant thicknesses” before ultraviolet irradiation. The carbon abundance in such “static” films is conserved, except for small photodesorption losses, ~10^−2^ to 10^−3^ CH_4_ photon^−1^ (*19*, *20*), because (unlike at Charon) no additional CH_4_ is condensed onto the films during ultraviolet processing. In a static experiment, relative abundances of methane and other C-bearing refractories approach steady-state values following irradiation to high doses due to near-equal photodestruction and reformation rates. However, conditions at Charon differ starkly from these “constant thickness” experiments because the continual methane condensation onto the surface during photolysis creates a “dynamic photochemistry” in which species concentrations do not converge to equilibrium values before being buried below the Ly-α absorption depth [photoactive layer (PAL)] by additional “fresh” methane.

Refractory concentrations in a dynamically photolyzed film are influenced by φ, which we define as the ratio of the methane accretion rate to the Ly-α flux (Fig. 1B), but this dependence has never before been characterized. Accretion can outcompete photodestruction in the case of high φ, where methane molecules become quickly buried below the PAL, inaccessible to the Ly-α photons. This PAL extends ~35 nm from the ice-vacuum interface and is the Ly-α optical penetration depth in solid methane (*21*). Conversely, at low φ, methane conversion to refractories might be more effective owing to the increased time for molecule-photon interactions available before burial beneath the PAL. In the “dynamic photolysis” experiments described in this paper, we have measured the dependence of photoproduct concentration on φ over Charon-relevant values, as informed by the exospheric model. We feed the conversion cross section from the experiments back into the exospheric model to constrain the photoproduct synthesis rate versus position and time on Charon’s surface.

## RESULTS

### Charon exosphere model

Charon orbits within Pluto’s outflowing methane cloud intercepting a peak flux of ~7.5 × 10^11^ CH_4_ m^−2^ s^−1^ on its leading hemisphere apex (*9*). Our exospheric model (*22*) approximates this Plutonian methane source to be constant and tracks the methane molecules following their initial impact. CH_4_ molecules transiently stick to Charon’s ~10- to 60-K surface and desorb stochastically at a temperature-dependent rate. Charon’s surface temperatures are modeled for a thermal inertia of 10 J m^−2^ K^−1^ s^−1/2^, an albedo of 0.3, and an emissivity of 0.9, as in the study of Grundy *et al.* (*3*). Most methane molecules desorbing from even the highest temperature locations do not exceed Charon’s escape velocity and become entrained in gravitationally bound trajectories, hopping across the surface in random directions (Fig. 1A, blue arrows) to generate a tenuous, collisionless exosphere. Following several such hops, these molecules often encounter frigid spots near Charon’s winter pole where they remain cryo-trapped until spring sunrise.

Cold locations on Charon therefore receive a continual supply of methane, but this cryo-trapping is concurrent with IPM Ly-α irradiation, as illustrated by the orange arrows in Fig. 1A. The IPM Ly-α flux at Charon was observed by the NH Alice instrument and estimated as ~140 R over the anti-Sun hemisphere or equivalently 3.5 × 10^11^ Ly-α photons m^−2^ s^−1^ (*23*). During accretion, IPM Ly-α continuously converts a fraction of the incoming methane molecules into higher-order hydrocarbons. A methane-rich frost containing these complex hydrocarbons accumulates atop the cold surfaces over the 124-year-long winter. Spring sunrise warms this polar frost and drives unprocessed methane back into the gas phase, resulting in an equinoctial surge in Charon’s global exospheric density. These semiannual surges are transient, each lasting only a few years of the 248 Earth-year orbital period. Methane released from the spring polar zone readsorbs onto the cooling autumn polar region as it recedes into winter darkness. This seasonal pole-to-pole transference of methane is an important new insight gleaned from the exospheric model. The reader is referred to Teolis *et al.* (*22*) for more details on the exospheric model, including an in-depth discussion of the size and formation rate of the polar methane cap under flash-freezing versus slow-accretion scenarios during the equinoctial exchange of methane between spring/autumn polar regions and its dependence on thermal inertia over a range of 2.5 to 40 J m^−2^ K^−1^ s^−1/2^. At low (2.5 J m^−2^ K^−1^ s^−1/2^) inertia, methane coming off the spring polar zone is “promptly” (over ~4 years) refrozen into a small polar cap at the rapidly cooling autumnal polar zone. At high (40 J m^−2^ K^−1^ s^−1/2^) inertia for which the autumnal polar zone cools more slowly, larger polar caps extending to lower latitudes form more gradually over several decades.

**Fig. 2.**
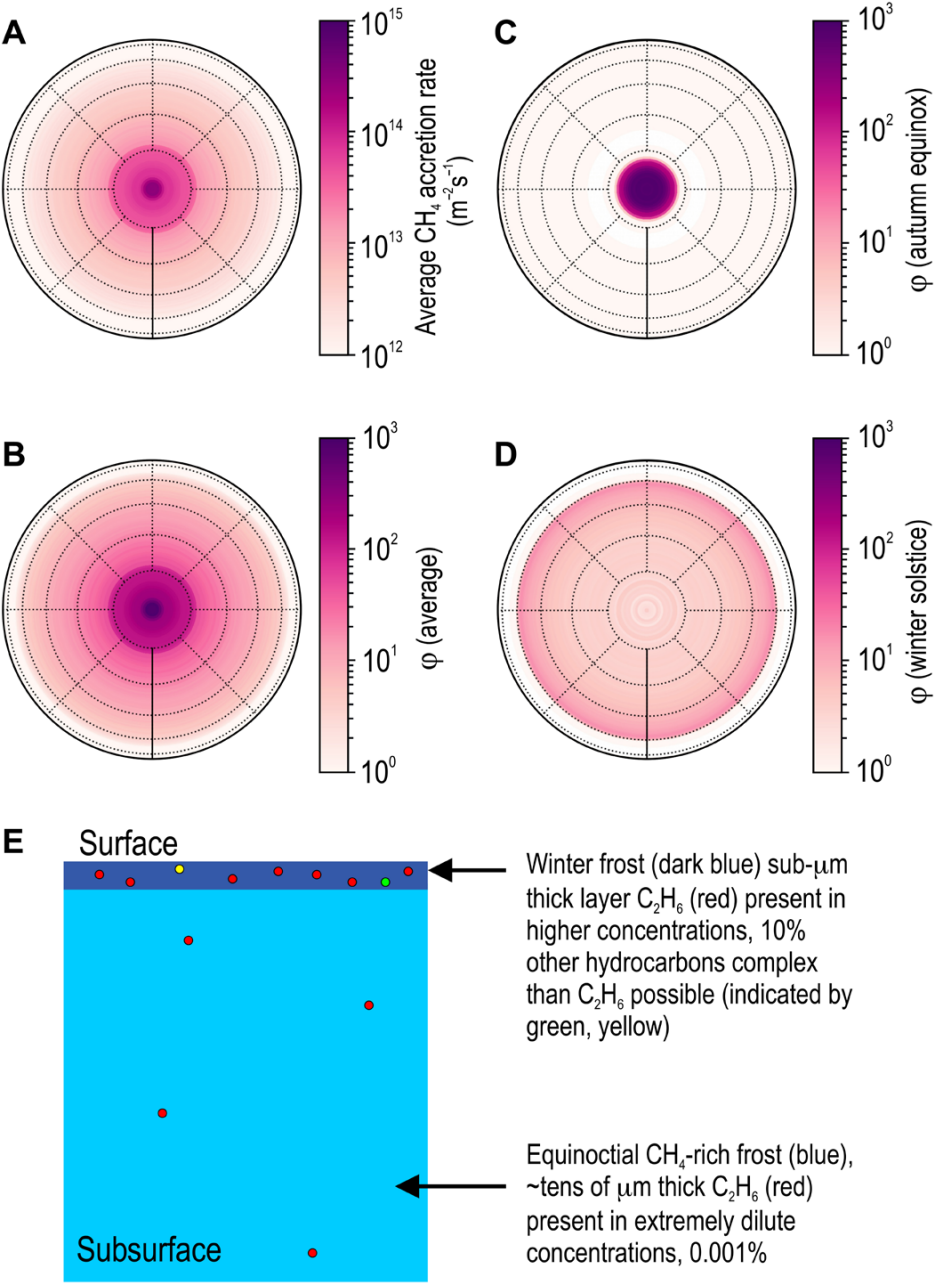
Charon’s seasonally varying φ maps. Modeled pole-centric maps of methane cold-trapping rate (**A**), and φ ratio, obtained by dividing the cold-trapping rate by the isotropic IPM Ly-α flux (**B**) over Charon’s winter hemisphere. The exosphere simulations track CH_4_ molecules as they ballistically migrate to polar “cryotraps” following initial impact (*9*). Seasonal variations in methane escape from Pluto’s atmosphere are ignored in these simulations. Both CH_4_ accretion rate and φ are time-averaged for several Pluto orbits at present-day precession and for an orbit with a ±90° offset in the solar longitude of perihelion relative to the present-day angle. Only the winter segment of the 248-year orbit where methane accretion rate values are positive is considered in the time average. Also shown are snapshot φ maps at autumn equinox (**C**) and winter solstice (**D**). The temperature in the polar zone >70° plummets quickly below 30 K as the hemisphere recedes into winter night to capture most of the methane desorbing from the opposite spring pole. This leads to rapid accretion of methane-rich polar cap, ~30 μm thick, within a year following autumn equinox. Methane from Pluto continues to accrete onto the cold pole forming a thinner (~0.3 μm) layer over thick equinotical frost over the 124-year winter (**E**). Very little methane is photolyzed during this high-φ accretion as indicated by the red dots in (E). More complex species result during low-φ photolysis indicated by multicolored dots present in the winter frost layer. Charon surface temperatures are modeled for thermal inertia of 10 J m^−2^ K^−1^ s^−1/2^ and albedo and emissivity values of 0.3 and 0.9, respectively. The annuli (dotted circles) are latitudes in 15° increments, while dotted lines are longitudes in 45° increments referenced from the prime meridian (solid line).

The exospheric simulations output time-dependent CH_4_ cold-trapping rates at all Charon locations while also accounting for the ballistic redistribution of Plutonian methane (*24*) and the seasonal pole-to-pole swapping. The latitudinal dependence of the methane accumulation rate, selectively time-averaged over a Pluto orbit at times when adsorption exceeds desorption, is shown in Fig. 2A. Surface methane coverage approach 10^24^ molecules per square meter at the pole, resulting in a frost several tens of micrometers thick over the course of a single winter. Low-latitude regions are depleted in methane relative to the poles owing to their higher surface temperatures [see the study of Grundy *et al.* (*3*); also see fig. S1 for evolution in surface temperatures at different latitudes over a Pluto orbit]. We also divide the time-averaged CH_4_ accretion rate by the IPM Ly-α flux to obtain average φ values on Charon’s surface. A pole-centric Charon average φ map is shown in Fig. 2B, along with snapshots near autumn equinox (Fig. 2C) and at winter solstice (Fig. 2D). The low latitude zones closer to the solar terminator experience a higher φ ~ 10 to 15 than the poles, owing to warmer temperatures that promote methane “hops” as opposed to the cold trapping where methane molecules stick to the polar surface. These maps (Fig. 2, A and B) show a sharp rise in methane accretion rate and φ north of 70°. With the onset of autumn, the temperature within this polar region plunges quickly below 30 K to capture a large fraction of the methane released into the exosphere from the opposite spring pole. A polar methane-rich frost accrues to several tens of micrometer thickness under conditions of extreme φ peaking at ~30,000, ~2 years before autumn equinox. Following this equinoctial pole-to-pole methane transfer, the rate of accretion slows down, decreasing φ to ~4, as shown in the winter solstice snapshot map (Fig. 2D). On average nearly ~75% of the global condensed methane is sequestered inside this polar zone. We determine through our dynamic photolysis experiments the fraction of the methane frost that is converted into refractory photoproducts by the IPM Ly-α and constrain its spatial distribution on Charon’ surface.

In addition to modeling conditions relevant to Charon’s present day orbit around the Sun, we have run also exospheric simulations at several solar longitudes of perihelion. Averaging these model results over perihelion longitude yields estimates of the cumulative distribution of photoproduct synthesis over geologic time scales over which Pluto’s eccentric orbit precesses. These “precession-averaged” exospheric models predict identical methane coverages, accretion rates, and photoproduct distributions in both the northern and southern hemispheres, as the seasonal dependence of the Pluto-Charon system’s heliocentric distance and solar insolation flux are averaged out. Pole-centric maps in Fig. 2 therefore represent polar conditions that emerge over the long 2.8-Ma precession cycle (*24*–*26*) but do not include effects from micrometeoroid gardening.

### Dynamic photolysis experiments

A subset of the model-predicted Charon φ values are well reproduced in our dynamic photolysis experiments. We condense methane films onto a quartz crystal microbalance (QCM) cooled to 10 K, the temperature at Charon’s winter pole (*3*). Methane accretion shifts the resonant frequency of the piezoelectric QCM, which is proportional to the areal mass (ng/cm^2^) of the condensate (*27*). The films were deposited at a constant rate in each experiment; the deposition rates were varied between ~10^16^ and 10^18^ CH_4_ m^−2^ s^−1^. Concurrent with methane deposition, the QCM is also exposed to Ly-α flux from our microwave discharge (MWD) lamp. Although MWD lamps are generally a broadband source in the vacuum ultraviolet, their spectral output can be tuned to maximize the flux at Ly-α relative to longer-wavelength H_2_ molecular emissions. Our MWD lamp operational conditions are adopted from the study of Chen *et al.* (*28*) to ensure high Ly-α output (> 75% total flux). The longer-wavelength H_2_ molecular emissions that make up ~25% of the spectral flux do not contribute significantly to the photodestruction as methane photoabsorption cross section > 130 nm is negligible (*29*). We measure the Ly-α flux using a 98% transparent Au grid placed between the MWD source and the QCM (see Materials and Methods for additional details on instrumentation). Typical Ly-α flux in our experiments is ~10^16^ photons m^−2^ s^−1^, in close agreement with the study of Chen *et al.* (*28*). We adjust the methane deposition rate relative to the Ly-α flux to achieve laboratory φ values representative of Charon.

**Fig. 3.**
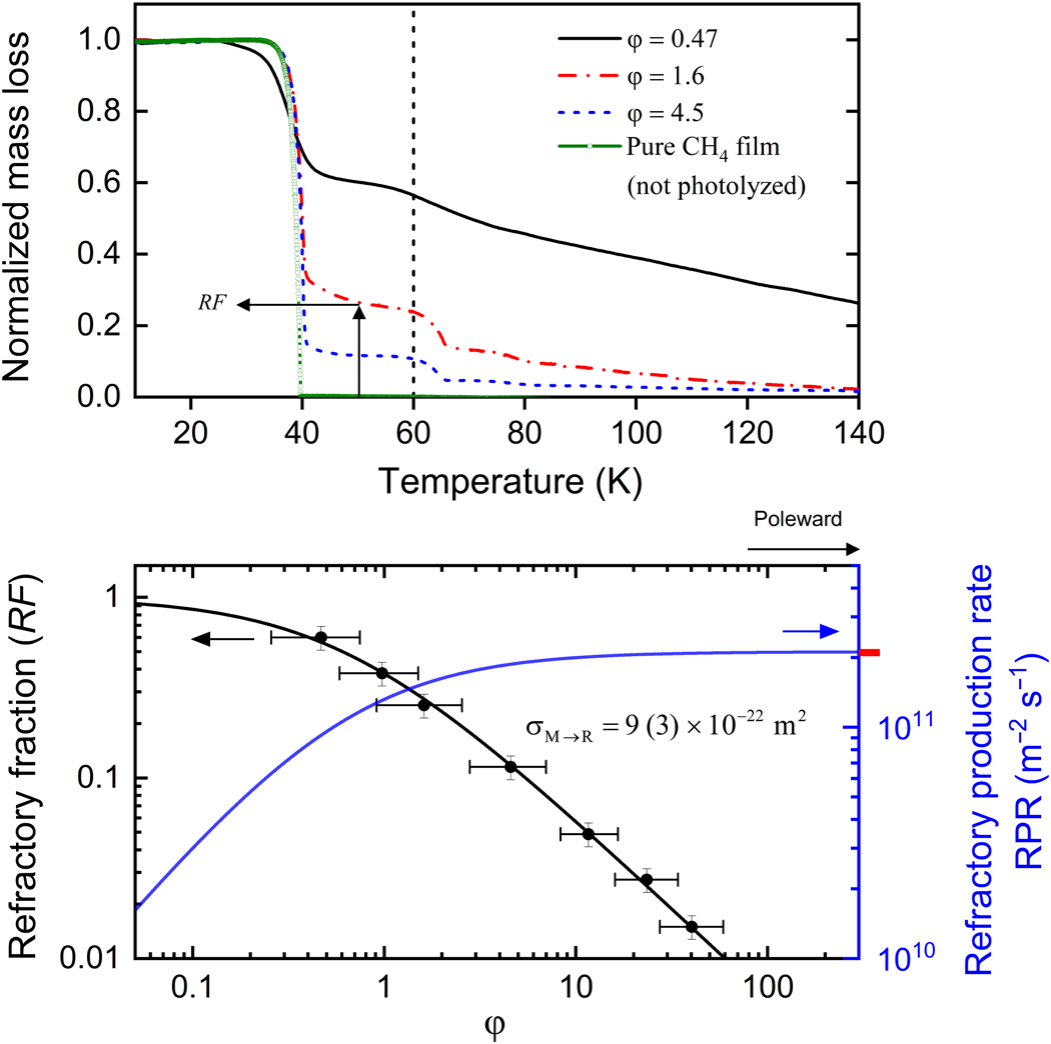
Refractory fraction *RF* synthesized in dynamically photolyzed CH_4_ films. Mass loss during controlled thermal desorption of Ly-α processed methane films photolyzed at different φ values are shown in the top panel. The mass loss is normalized to total methane mass. The first decrease in film mass at ~40 K is due to desorption of the methane molecules unaffected by Ly-α photons. The second decrease at ~65 K is from C_2_H_6_ desorption. The mass fraction retained at 50 K (top, arrows) belongs to photolyzed refractories more complex than methane. We plot the refractory fraction against φ (black circles) in the bottom panel. Methane films photolyzed at higher φ contain fewer refractories. The solid black curve is a model fit (Eq. 2B) to the *RF* versus φ data, which gives the methane-to-refractory conversion cross section σ_M → R_. The solid blue curve shows the dependence of the refractory production rate (RPR) on φ. RPR, which is *RF* × *F*_M_, increases steadily with φ; the red dash indicates a maximum conversion rate.

A fraction of the condensing methane is converted into heavier refractories by the Ly-α photons, resulting in the accumulation of a photolyzed mixture of CH_4_ and higher-order hydrocarbons on the QCM surface. Once a desired film mass is achieved, the photolyzed films are heated to higher temperatures at 1.5 K min^−1^. This heating phase of the experiments mimics the warming of Charon’s winter polar zone as it emerges into sunlight with the approaching spring equinox. The QCM continually monitors the mass loss during the thermal desorption of methane and refractory photoproducts. The top panel of Fig. 3 shows the desorption profiles of the methane films photolyzed at three different φ values, normalized by the total methane equivalent mass of the condensed film. Intact CH_4_ molecules unaffected by Ly-α photons desorb at ~40 K, as observed for an unirradiated methane film. The second mass loss at ~65 K is mainly due to C_2_H_6_ desorption. Higher-order refractories continue to desorb steadily past 65 K.

The mass fraction retained on the QCM at 50 K belongs to the heavier photoproducts complex than CH_4_. Photolysis at a low φ of 0.47 transforms ~60% of the accreting methane into higher-order hydrocarbons. The refractory fraction *RF* drops to ~2% in methane film photolyzed at φ = 23. The dependence of *RF* on φ is shown in the bottom panel of Fig. 3. For simplicity, *RF* denotes the fractional abundance of all refractory photoproducts different from CH_4_, ignoring the compositional complexity of the higher-order refractories. Refractory photoproducts are synthesized in increasingly dilute concentrations when methane films are photolyzed at higher φ values. As a result, Charon’s polar frost photolyzed at a high φ ranging from ~30,000 (near equinox) to 4 (solstice) are methane rich, containing photoproducts at extremely dilute concentrations ranging from 0.001 (equinoctial frost) and 15% (post-equinox layer), depending on the depth in the frost layer and the instantaneous φ at the time of deposition.

Methane conversion to refractories occurs most efficiently in the PAL, which is the ~35-nm photoabsorption depth below the top surface (*20*). In dynamic photolysis, methane photodestruction competes with accretion of fresh methane that is continually added to the PAL, which gradually buries methane and refractories until they are beyond the optical penetration depth (PAL tracks the “advancing” surface as the film thickness grows). Heavier refractories and unprocessed methane accumulate beneath the PAL and continually accrete atop the QCM or over Charon’s cold-surface locations (Fig. 1B).

The time evolution of the methane abundance in the PAL (η) is determined by (i) the methane arrival rate *F*_M_, (ii) the CH_4_-to-refractory conversion rate in the PAL, and (iii) the burial rate of the photolyzed methane-refractory mixture beneath the PAL (Fig. 1B). These processes are captured by the rate equation

$$\frac{d\eta }{\mathrm{dt}}=F_{M}-\sigma_{M\to R}\;\rho \;d_{\text{PAL}}F_{\mathrm{Ly}\alpha }-\gamma F_{M\;}$$ (1A)

where σ_M → R_ is the methane-to-refractory conversion cross section, ρ is the density of the photolyzed frost, *d*_PAL_ is the Ly-α penetration depth in methane ice, *F*_Lyα_ is the Ly-α flux, and γ is the relative methane concentration with respect to the total methane + refractory abundance in the PAL. Equation 1A in terms of φ = *F*_M_/*F*_Lyα_ reads

$$\frac{1}{F_{M}}\frac{d\eta }{\mathrm{dt}}=1-\frac{\sigma_{M\to R}\;\rho \;d_{\text{PAL}}}{\varphi }-\gamma$$ (1B)

A constant CH_4_ abundance is achieved in the PAL when methane depletion due to photodestruction and seepage to subsurface below the PAL is balanced by the methane condensation rate. Solutions to Eq. 1 gives methane and refractory fractions in the PAL in the limit of constant φ

$$\gamma =1-{\left(\xi \varphi +1\right)}^{-1}$$ (2A)

$$\mathrm{RF}={\left(\xi \varphi +1\right)}^{-1}$$ (2B)

where ξ = (σ_M → R_ ρ_M_ *d*_PAL_)^−1^ and ρ_M_ is the density of solid methane. The inverse dependence of refractory concentration on φ is clear in Eq. 2B. The previously photolyzed material buried beneath the PAL (Fig. 1B) is mostly shielded from Ly-α photons and therefore retains to good approximation the relative methane and refractory concentrations as per Eq. 2.

The conversion cross section σ_M → R_ is the only free parameter in Eq. 2B. We constrain σ_M → R_ to 9 (± 3) × 10^−22^ m^2^ through the fit of Eq. 2B to the *RF* versus φ data (black curve in the bottom panel of Fig. 3). This conversion cross section is smaller than the 1.4 × 10^−21^ m^2^ photoabsorption cross section reported by Cruz-Diaz *et al.* (*10*), likely due to enhanced geminate recombination in the solid phase (*30*). The surrounding lattice in the solid phase can collisionally quench the excess kinetic energy carried by the dissociated photofragments and trap them within the lattice “cage” to reconstitute the parent species. The cage effect is negligible in the gas phase, owing to low density. A constant σ_M → R_ emerging from the robust fit of Eq. 2B to the *RF* versus φ dataset over a wide range of φ values suggests that “cage effect” is not significantly diminished in conditions of slow accretion (φ ~ 0.5) compared to the high-φ cases. The pliability of the lattice cage is unchanged over the range of φ measured in our dynamic photolysis experiments. Our conversion cross section is larger than the 0.7 × 10^−22^ to 2 × 10^−22^ m^2^ photodestruction cross section (*13*, *18*), possibly because it is derived from thermal desorption datasets that include not only photoconversion but enhancements in the refractory concentrations during the heating phase from radicals reacting at temperatures >10 K (*31*–*33*). In addition, previous estimates derived from the decay of the methane infrared absorption with photon dose did not fully account for optical interference effects (*34*), which could contribute to the difference with our values as well.

Equation 2B predicts the relative refractory abundance in a dynamically photolyzed methane film where φ is held constant in a controlled experiment, but φ on Charon’s surface changes drastically over a Pluto orbit because of exospheric surges in global methane density at equinox (*22*) and changes in cold trapping efficiency due to the seasonal surface temperature cycle. A nonconstant φ scenario requires numerically solving for Eq. 1 across Charon’s surface. We therefore incorporate σ_M → R_ obtained from the experiments into the exosphere code to estimate the photoproduct synthesis at all locations on Charon’s surface.

## DISCUSSION

### Applications to Charon

**Fig. 4.**
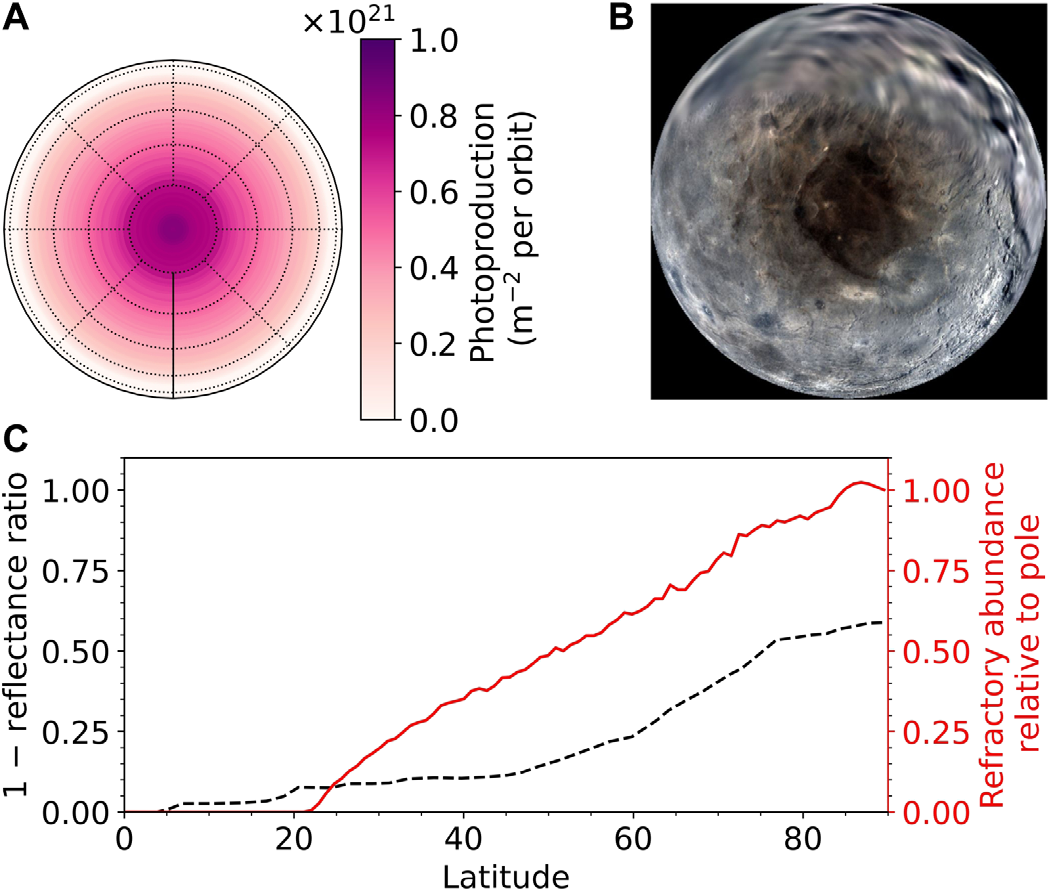
Photoproduct distribution on Charon imprinted by IPM Lyman-α. (**A**). Pole-centric view of the time- and precession-averaged refractory abundance (per orbit), which steadily increases with latitude to a polar maximum of ~80 ML. The spatial dependence of the refractory abundance is remarkably consistent with the latitudinal gradient of the albedo captured in the MVIC + LORRI composite map [from Schenk *et al.* (*47*)] shown in (**B**). The abundance, normalized to the polar maximum, is compared to 1 − *R* in (**C**). *R* is the reflectance ratio [Figure 2D of the study of Grundy *et al.* (*3*)] obtained by dividing latitudinally averaged brightness in the observed image by a modeled image assuming a uniform albedo.

The distribution of photoproducts over Charon’s surface, produced over a Pluto orbit (Fig. 4A), falls off smoothly with distance from the pole, in accordance with the duration of the polar night versus latitude. The explanation is that heavier hydrocarbons are produced over the entirety of polar night at a near-constant rate of ~2 × 10^11^ m^−2^ s^−1^, which is the maximum possible in the limit of high φ (Fig. 3, bottom). The exosphere model shows that despite the expected extreme variability of the CH_4_ condensation rate (*22*), φ never drops below ~4 over the polar winter. Methane accrues onto the night zone at least an order of magnitude faster than it can be photolyzed, thereby maintaining a high methane concentration within and below the PAL and the subsurface beneath (Fig. 1B). At the pole, the dynamic photolysis of methane by the IPM Ly-α photons results in 80 monolayers (ML) (with 1 ML defined as 10^19^ molecules/m^2^) of complex hydrocarbons over the course of a single Charon winter. Methane spends less time on the surface at lower latitudes where the polar night is shorter, and therefore, photolysis has less time to produce complex hydrocarbons at these latitudes. Thus, the photoproduct abundance thins gradually with decreasing latitude dropping to nearly half the polar value at 45°. This latitudinally varying refractory distribution, consistent with Charon’s albedo gradient (Fig. 4, B and C), is a notable improvement over the ad hoc approximation of a uniform 155-ML-thick refractory coverage north of 45° (*3*). Topographic shadowing and local variations in thermal inertia can affect the predicted smooth fall-off in refractory distribution. Such second-order refinements to the thermal and exospheric modeling will be addressed in a future study. Globally, only ~10% of the methane arriving from Pluto is converted to complex species, smaller than the previous estimate of 21% (*3*). A lower cross section (9 × 10^−22^ m^2^ versus 14 × 10^−22^ m^2^) combined with inefficient photoconversion of polar methane during rapid accretion (high φ) over a much smaller zone (north of 70° versus the expanded 45°) explains our reduced estimate.

The surface methane distribution averaged over time and precession (Fig. 2A) differs substantially from that of the photoproducts as it exhibits a sharp step at ~75^o^ latitude. These steps are the methane polar caps, “flash-frozen” over ultrashort ~1 Earth year surges of exospheric gas density near the autumnal equinoxes as frozen methane rapidly sublimes from the opposite (spring) polar zone (*22*). These rapidly condensed polar caps are several tens of micrometers thick, far beyond the ~35-nm optical depth over which Ly-α photolysis can take place. Over the long polar winter, an additional several hundred nanometers of methane slowly accrete over the 1/σ_M → R_*F*_Lyα_ ~ 100 Earth years photoconversion time, an accretion rate that is still an order of magnitude faster than the topmost ~35 nm can be photolyzed. Charon’s photolysis therefore takes place largely in a “high-φ” regime, resulting in highly dilute photoproduct concentrations in the polar night zone surface frost with maximum concentrations of only ~15%.

**Fig. 5.**
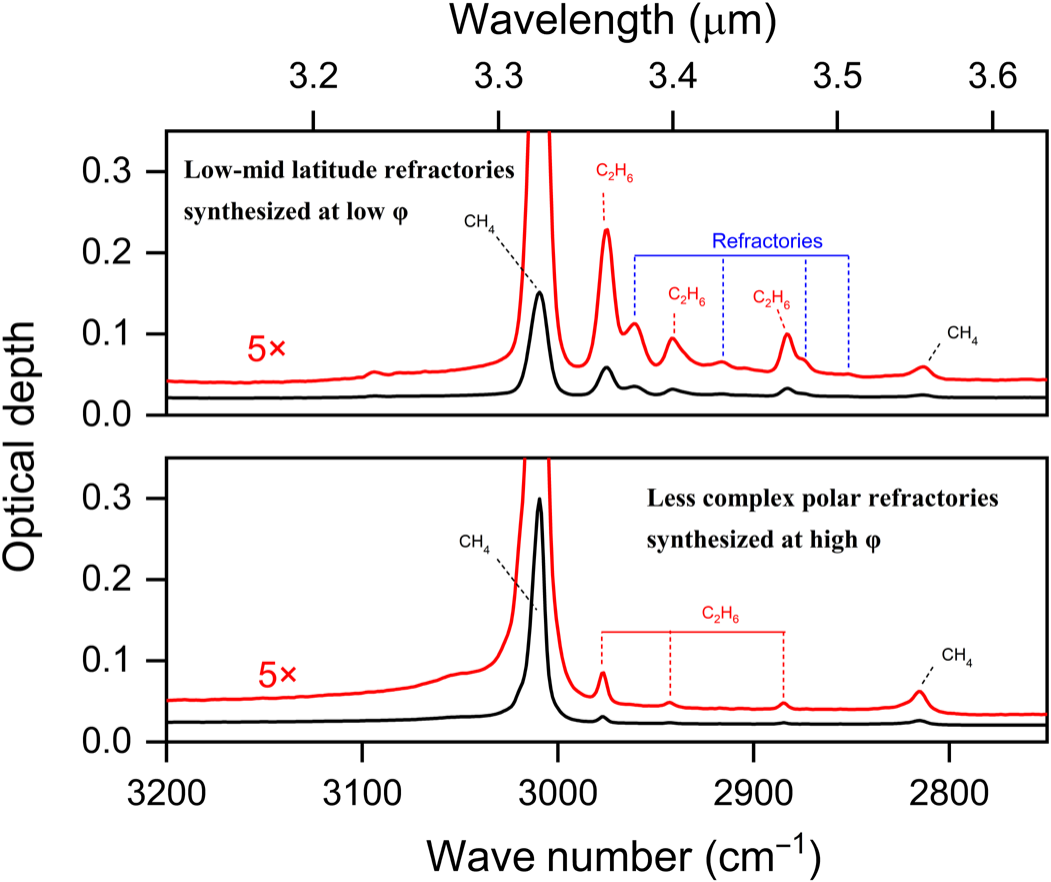
Infrared spectra of dynamically processed films. The top panel shows that a diverse, complex family of heavier hydrocarbons are synthesized in conditions of low φ. In contrast, high-φ photolysis result in formation of mainly ethane (bottom). These films were photolyzed at 10 K. Ethane therefore would be the dominant constituent of the polar refractories, while Charon’s lower latitudes would host increasingly complex refractories. Detailed assignments of absorption bands between 3 and 3.6 μm are given by Carrascosa *et al.* (*18*) and reference therein.

The photoproduct composition at such low concentrations is predominantly ethane because the PAL has little time to undergo photolysis before being buried by accreting methane beneath the PAL (Fig. 1B). Therefore, dynamic photolysis (characterized by the φ parameter) controls not just the refractory distribution on Charon but also the composition of its refractories. We compare infrared spectra of methane films of similar thickness photolyzed at 10 K with low (=1.6) and high (=23) φ in Fig. 5. The two films differ significantly in composition. The low-φ film shows a diverse set of refractory photoproducts more complex than ethane, as indicated by the several absorption bands near the 3-μm spectral region (top). These heavier refractories resist thermal desorption, resulting in the protracted mass loss at temperatures >65 K, especially noticeable in the film photolyzed at φ = 0.47 (top of Fig. 3). Assignment of the absorptions to specific hydrocarbons detected in the low-φ film is provided by Carrascosa *et al.* (*18*) and references therein.

By comparison, the high-φ film only shows infrared absorptions corresponding to C_2_H_6_. More complex hydrocarbons may be present at abundances below our detection threshold. The dilution of photoproducts by rapid methane accretion and rapid burial of secondary photoproducts (i.e., ethane) beneath the PAL in the high-φ films curb the production of the ethyl radicals needed to form increasingly complex refractories. Hence, C_2_H_6_ emerges as the dominant photoproduct at Charon. This is especially true close to the poles, where the methane polar caps, tens of micrometers thick, are formed in only ~3 Earth years, so short a time that photoproduct concentrations within these thick frost layers are as low as ~0.001%. Photoproduct concentration, composition, and complexity are a function of time, latitude, and depth within the surface frost, controlled by the seasonally varying φ.

Ethane in the polar frost has interesting color implications. Spring sunrise drives off the unprocessed methane as the pole warms to 60 K, leaving behind the ~80-ML-thick layer that is mostly ethane. While more rigorous measurements of optical constants of solid ethane in the visible spectrum are needed, limited data suggest that it is colorless (*35*) and therefore would not contribute to the red color of Charon’s pole. However, solar wind impinging on the summer pole over ~30 years (the time required for surface temperature to warm up to 60 K) can further process the IPM Ly-α–cooked ethane frost, forming complex refractories needed to impart the red color. Assuming a cross section of ~10^−19^ m^2^ (typical for ion-induced radiolysis), 1-keV solar wind impinging at a 10^10^ m^−2^ s^−1^ flux (*36*, *37*) can generate about 50 ML of more complex refractories in 30 years. The kilo–electron volt proton transfers ~10 eV/nm as it traverses through the ethane frost (*38*), resulting in a high density of ionizations in the vicinity of the ion track. Ethyl radicals generated in close proximity in the track (unlike diffuse excitations induced by the solar and IPM Ly-α photons simultaneously impinging onto the summer pole) can react to form more complex species and possibly redder refractories. At 60 K, ethane thermally sublimates on the order of a month, but three decades of solar wind processing could produce higher-order, carbon-enriched refractories (*39*–*41*) that resist sublimation and are further radiolyzed over the duration of the Charon day.

## SUMMARY AND OUTLOOK

The photoproduct distribution map produced by combining an exosphere model with CH_4_ photolysis experiments is consistent with the poleward decline in Charon’s albedo visible in New Horizons imagery. Our efforts elucidate the importance of the dynamic photolysis and the flux ratio φ between condensing methane and IPM Ly-α photons in determining abundance, compositional complexity, and their latitudinal variation across Charon’s surface. High-φ photolysis converts the polar methane into ethane, which is unlikely to contribute to the reddish hue. However, solar wind could process the ethane frost during the summer to produce redder, more complex hydrocarbons that impart Charon its unique polar color. Furthermore, reduced solar wind flux at mid-latitudes may process hydrocarbons to a different degree of complexity and color resulting in different darkening agents as noted by Protopapa *et al.* (*11*). Our results constrain the abundance of the refractories generated by IPM Ly-α photons on Charon’s surface. Optical constants of the refractories in the visible and near-infrared spectral region and their dependence on φ, along with estimates for the color of solar wind–processed ethane ices, are needed to construct a photometric model of Charon for apposite comparison to New Horizon images.

Dynamic photolysis may not be unique to Charon but rather a common occurrence in the outer Solar System. Fallout from cryovolcanic plumes on many Ocean Worlds like Enceladus, Europa, or Triton, as well as fresh coating of the Saturnian satellites with icy grains sourced from the E-ring, could be processed in a manner similar to Charon’s methane frost. The flux ratio of accruing species relative to energetic photons and particles can be a key determinant of the observed abundances and complexity of radiolytic/photolytic by-products. For instance, the synthesis of solid ozone, observed in several Jovian (*42*) and Saturnian satellites (*43*), has been demonstrated when water ice is subject to particle irradiation during accretion (*44*). The framework of dynamic photolysis established from this study of Charon can be adapted to better understand the energetic processing of accreting ices in various dynamic extraterrestrial environments.

## MATERIALS AND METHODS

### Charon thermal model

To account for the effect of Charon’s surface temperature versus latitude, longitude, and time, we have embedded a thermal model into our exospheric model. The thermal modeling approach is identical to that used by Grundy *et al.* (*3*), following the long-established method described by Spencer *et al.* (*45*). Following the same approach, we solve a time-dependent heat diffusion equation for temperature *T* in three dimensions across the planetary body (latitude, longitude, and depth). The equation takes into account the surface solar insolation source and its variation according to Charon’s heliocentric distance, as well as the solar zenith angle versus local time, latitude, and solar declination angle. We consider as an approximation a uniform surface bolometric bond albedo of 0.3, such that 70% of the incident solar energy is absorbed by the surface. We assume a surface emissivity of unity, such that the surface radiates heat back to space at a rate given by σ*T*^4^, with σ the Stephan-Boltzmann constant. The heat diffusion constant *D* = (*I*/*C* × ρ)^2^ = 3.9 × 10^−11^ m^2^/s for a thermal inertia = 10 J m^−2^ K^−1^ s^−1/2^, where *C* = 800 J kg^−1^ K^−1^ and ρ = 500 kg/m^3^ are the surface heat capacity and mass density, respectively (*3*). The model results shown in fig. S1 for thermal inertia = 10 J m^−2^ K^−1^ s^−1/2^ are as expected identical to those (*3*) for the Pluto-Charon system’s present-day longitude of perihelion.

### Instrumentation details for the dynamic photolysis experiments

The methane photolysis experiments were performed in an ultrahigh vacuum (UHV) chamber cryo-pumped to a base pressure of ~10^−10^ torr. A ~6-MHz QCM embedded onto a Cu sample holder is bolted to the terminal end of a 4-K closed-cycle cryostat. The cryostat is mounted vertically on a rotatable stage to place the QCM at the center of the UHV chamber. The rotating stage enables the QCM to face several instruments installed onto the multiple ports on the chamber wall.

The QCM is cooled to 10 K before exposing it to a collimated flux of methane molecules from a microcapillary array doser. The resonant frequency of the QCM decreases in response to the mass loading during methane deposition; the change in the frequency is proportional to the column density (molecules cm^−2^) of the thin methane films. Collimated deposition of methane in our experiments is different from “omnidirectional” freezing occurring on Charon, but differences in film microstructure (phase, porosity) between deposition techniques (*46*) are not likely to affect Ly-α photochemistry. During deposition, the CH_4_ films are irradiated with Ly-α photons emanating from the microwave-discharge lamp. The operating conditions of the lamp are chosen to maximize the Ly-α flux in their spectral output. The methane deposition rates are varied relative to the near-constant Ly-α flux to change φ over nearly two orders of magnitude. Photolyzed films are heated at a controlled rate of 1.5 K/min, while the mass loss due to desorption is continuously monitored with the QCM. The mass fraction of methane and higher-order refractories in the photolyzed films presented in Fig. 3 are determined from the programmed thermal desorption experiments.

The front face of the QCM has a specular gold coating that enables reflectance spectroscopy on thin ice films deposited onto the QCM. Infrared spectra of the dynamically photolyzed methane films shown in Fig. 5 were collected using an iS50 Fourier transform infrared spectrometer. The spectra, obtained at 2-cm^−1^ resolution at 35° incidence, are expressed in optical depth units, −ln(*R*/*R*_0_), where *R* and *R*_0_ are reflectances of the photolyzed methane films and the Au substrate coating the QCM, respectively.
