# Supplementary material for: Charon’s refractory factory
Source: Sci Adv. 2022 Jun 17;8(24):eabq5701. doi: 10.1126/sciadv.abq5701 (PMC9205591; doi:10.1126/sciadv.abq5701)
Supplement: Supplementary file 1 — Fig. S1 Table S1 [file sciadv.abq5701_sm.pdf]

Science Advances
AAAS

Supplementary Materials for

Charon's refractory factory

Ujjwal Raut *et al.*

Corresponding author: Ujjwal Raut, uraut@swri.edu

Sci. Adv. **8**, eabq5701 (2022)
DOI: 10.1126/sciadv.abq5701

This PDF file includes:

Fig. S1
Table S1

Supplementary Materials

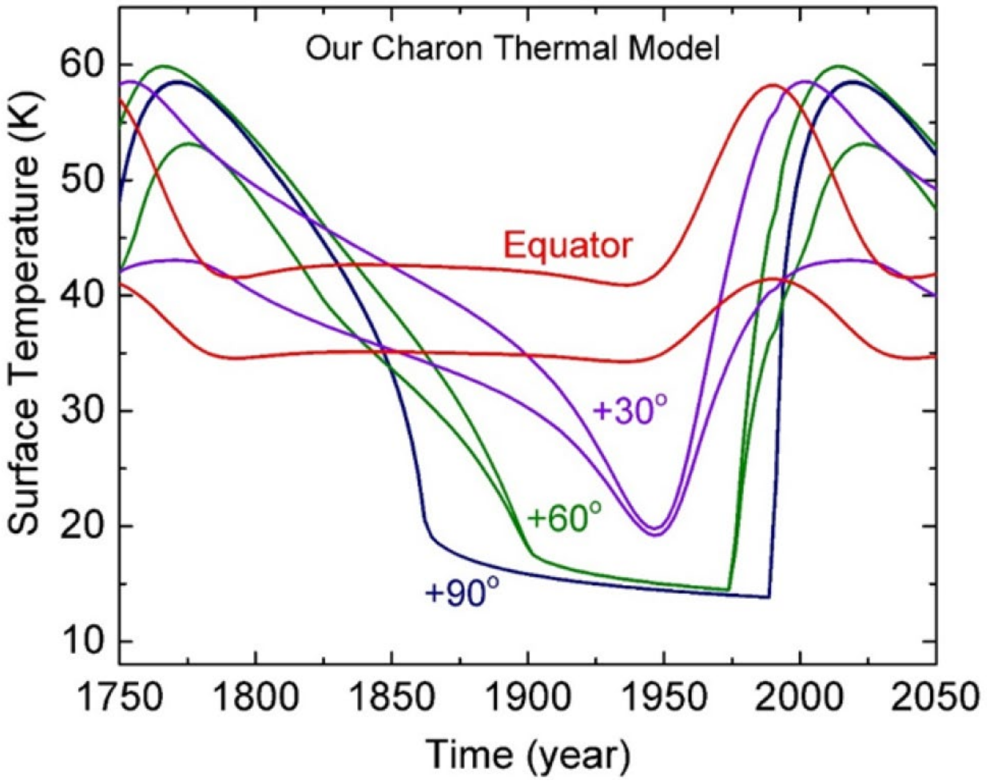

Fig. S1. Modeled Charon surface temperature evolution over a Pluto-Charon year at several latitudes, with the bands showing the predicted diurnal range for present day longitude of perihelion.

Table S1 showing data corresponding to pole-centric figures of methane accretion rate, average phi and autumn equinox and winter solstice snapshots (Fig. 2A-D) and the photoproduct distribution (Fig. 4A).

Latitude (deg)	F2A: Average CH4 Accretion Rate (m-2 s-1)	F2B: phi (Average)	F2C: phi (Autumn Equinox)	F2D: phi (Winter Solstice)	F4A: Photoproduction (m-2 per orbit)
89.55	3.05E+14	871.48	840.4485	4.58	8.22E+20
88.65	2.98E+14	850.41	839.0875	4.25	8.29E+20
87.75	2.96E+14	844.91	805.9645	2.74	8.37E+20
86.85	2.99E+14	853.25	794.2525	2.56	8.41E+20
85.95	1.98E+14	566.47	831.4297	2.16	8.37E+20
85.05	1.23E+14	351.11	842.4375	3.89	8.25E+20
84.15	8.00E+13	228.70	746.7268	2.98	8.06E+20
83.25	7.42E+13	212.09	789.2408	2.05	7.79E+20
82.35	7.57E+13	216.17	644.4438	3.56	7.71E+20
81.45	7.15E+13	204.32	484.9618	3.39	7.63E+20
80.55	6.13E+13	175.16	335.2079	3.67	7.47E+20
79.65	5.61E+13	160.23	178.3808	3.04	7.55E+20
78.75	5.32E+13	151.99	163.1795	4.19	7.47E+20
77.85	4.63E+13	132.31	64.2181	4.47	7.39E+20
76.95	4.60E+13	131.29	4.9721	2.74	7.43E+20
76.05	4.58E+13	130.88	0.6396	3.20	7.28E+20
75.15	4.35E+13	124.29	0.3615	3.53	7.32E+20
74.25	3.98E+13	113.83	0.1587	3.30	7.20E+20
73.35	3.60E+13	102.81	0.0428	3.41	7.04E+20
72.45	3.14E+13	89.84	0.0006	3.61	7.08E+20
71.55	1.55E+13	44.18	0.0000	3.72	6.53E+20
70.65	1.57E+13	44.90	0.0000	3.95	6.61E+20
69.75	1.40E+13	39.93	0.0000	4.00	6.42E+20
68.85	1.36E+13	38.79	0.0000	3.84	6.14E+20
67.95	1.18E+13	33.62	0.0000	3.89	6.10E+20
67.05	1.11E+13	31.76	0.0000	3.98	5.91E+20
66.15	1.02E+13	29.04	0.0000	3.56	5.67E+20
65.25	9.44E+12	26.98	0.0000	3.70	5.67E+20
64.35	1.04E+13	29.73	0.0000	3.89	5.79E+20
63.45	8.56E+12	24.45	0.0002	3.87	5.44E+20
62.55	8.66E+12	24.74	0.0001	4.03	5.44E+20
61.65	7.69E+12	21.96	0.0002	3.75	5.24E+20
60.75	7.08E+12	20.24	0.0003	3.73	5.12E+20
59.85	7.17E+12	20.50	0.0002	4.27	5.05E+20
58.95	6.94E+12	19.83	0.0002	3.80	5.09E+20
58.05	6.81E+12	19.46	0.0003	4.55	4.89E+20
57.15	5.76E+12	16.45	0.0004	4.45	4.77E+20
56.25	5.68E+12	16.24	0.0004	4.01	4.58E+20
55.35	5.15E+12	14.73	0.0004	4.62	4.50E+20
54.45	5.29E+12	15.12	0.0004	5.05	4.50E+20
53.55	5.08E+12	14.52	0.0004	4.76	4.34E+20
52.65	4.72E+12	13.49	0.0005	5.34	4.26E+20
51.75	4.42E+12	12.63	0.0005	5.44	4.11E+20
50.85	4.21E+12	12.03	0.0006	5.22	4.19E+20
49.95	4.35E+12	12.44	0.0006	5.88	3.99E+20
49.05	4.15E+12	11.87	0.0007	5.55	3.95E+20
48.15	4.03E+12	11.51	0.0007	5.85	3.78E+20
47.25	3.44E+12	9.82	0.0008	6.48	3.63E+20
46.35	3.47E+12	9.92	0.0007	6.27	3.57E+20
45.45	3.16E+12	9.04	0.0007	6.69	3.44E+20
44.55	3.41E+12	9.75	0.0007	6.70	3.43E+20
43.65	2.86E+12	8.18	0.0008	8.02	3.22E+20
42.75	2.80E+12	8.01	0.0008	7.56	3.09E+20
41.85	2.62E+12	7.49	0.0007	7.80	3.15E+20
40.95	2.81E+12	8.04	0.0007	9.16	3.09E+20
40.05	2.41E+12	6.90	0.0008	8.78	2.89E+20
39.15	2.41E+12	6.87	0.0008	9.66	2.83E+20
38.25	2.17E+12	6.21	0.0007	9.75	2.79E+20
37.35	2.33E+12	6.65	0.0008	10.45	2.72E+20
36.45	1.98E+12	5.67	0.0008	11.11	2.50E+20
35.55	1.87E+12	5.34	0.0008	12.38	2.34E+20
34.65	1.77E+12	5.05	0.0008	13.13	2.28E+20
33.75	1.94E+12	5.54	0.0008	14.21	2.21E+20
32.85	1.61E+12	4.59	0.0009	16.15	2.03E+20
31.95	1.48E+12	4.24	0.0009	16.24	1.87E+20
31.05	1.47E+12	4.21	0.0008	13.79	1.81E+20
30.15	1.28E+12	3.66	0.0009	8.46	1.64E+20
29.25	1.25E+12	3.57	0.0009	3.27	1.49E+20
28.35	1.16E+12	3.31	0.0008	0.27	1.37E+20
27.45	9.87E+11	2.82	0.0008	0.02	1.17E+20
26.55	9.16E+11	2.62	0.0009	0.01	1.05E+20
25.65	7.11E+11	2.03	0.0009	0.01	8.53E+19
24.75	6.51E+11	1.86	0.0009	0.00	7.16E+19
23.85	4.50E+11	1.28	0.0009	0.00	4.87E+19
